# Specialized inpatient treatment for young people with early psychosis: acute-treatment and 12-month results

**DOI:** 10.1007/s00406-022-01379-8

**Published:** 2022-02-09

**Authors:** Stefan Siebert, Karolina Leopold, Johanna Baumgardt, Laura-Sophie von Hardenberg, Eva Burkhardt, Andreas Bechdolf

**Affiliations:** 1 Department of Psychiatry, Psychotherapy and Psychosomatic Medicine Incorporating FRITZ and soulspace, Vivantes Hospital Am Urban and Vivantes Hospital Im Friedrichshain, Charité-Universitätsmedizin, Dieffenbachstraße 1, 10967 Berlin, Germany; 2 Department of Psychiatry and Psychotherapy, University Hospital Cologne, Cologne, Germany; 3 Department of Psychiatry and Psychotherapy, Centre for Psychosocial Medicine, University Medical Centre Hamburg-Eppendorf, Hamburg, Germany; 4 Orygen, Parkville, VIC Australia

**Keywords:** Early intervention, Early psychosis, Psychosis, Inpatient treatment, Psychotherapy

## Abstract

The objective of the study was to investigate the development of clinical outcomes of young people with early psychosis in a specialized inpatient treatment and assess the feasibility of such an intervention in an inpatient setting. The study was a prospective cohort study of patients with early psychosis treated at the specialized inpatient treatment “Fühinterventions-und Therapiezentrum, FRITZ” (early intervention and therapy center) in Berlin, Germany. The primary outcomes were attitudes towards psychiatric medication and patient satisfaction with treatment after 6 weeks. Secondary outcomes were clinical symptoms, functioning, remission, recovery, all-cause treatment discontinuation, and rehospitalisation at 6 and 12 months after inpatient treatment. We recruited 95 inpatients with early psychosis. Attitudes towards psychiatric medication (Δ_6weeks_ = 3.00, *d*_6weeks_ = 0.55; Δ_6mo_ = 2.15, *d*_6mo_ = 0.35; Δ_12mo_ = 3.03, *d*_12mo_ = 0.52) and patient satisfaction (Δ_6weeks_ = 0.21, *d*_6weeks_ = 0.40; Δ_6mo_ = 0.32, *d*_6mo_ = 0.43; Δ_12mo_ = 0.13, *d*_12mo_ = 0.17) changed with medium effect sizes at six weeks up to a 6- and 12-month follow-up. Clinical outcomes changed significantly with medium-to-large-effect sizes over 12 months CGI_Δ12mo_ = 1.64, *d*_12mo_ = −1.12; PANSS total_Δ12mo_ = 20.10, *d*_12mo_ = −0.76; GAF_Δ12mo_ = 19.58, *d*_12mo_ = 1.25). The all-cause treatment discontinuation rate was 13.69% (*n* = 13) at a 6-month and 35.79% (*n* = 34) at a 12-month follow-up. The rehospitalization rate was 30.53% (*n* = 29) at a 6-month and 43.16% (*n* = 41) at a 12-month follow-up. Patients with specialized inpatient treatment for early psychosis showed improvements in attitude towards psychiatric medication, patient satisfaction, symptoms, and functioning for up to 12 months.

**Trial registration**: DRKS00024351, 2021/02/11 retrospectively registered.

**Supplementary Information:**

The online version contains supplementary material available at 10.1007/s00406-022-01379-8.

## Introduction

Psychosis is a chronic disease in most cases that can have devastating consequences for individuals, families, and societies [1, 2]. Studies have shown that the earlier people with psychotic disorders receive treatment, the more they benefit in terms of recovery, functioning, and rehospitalisation [3]. As a result, specialized early treatment interventions were developed to target the early phase of psychosis (EP). EP is defined as the 5 years after the onset of the first psychotic episode, a critical period in which fewer confounding factors, such as prolonged medication exposure and chronicity [4, 5], exist. In recent decades, numerous worldwide clinical and scientific projects have been carried out within the field of EP [6–8]. As the diagnostic criteria of different psychotic disorders overlap and an accurate diagnosis in many cases can only be assigned throughout the course of an illness, EP include other psychotic disorders besides schizophrenia. This especially applies to cases involving frequent substance use, where accurate assignment of diagnosis is only possible over a long-term period. Therefore, diagnoses often change throughout illness trajectory [9, 10]. Research has shown that diagnostic changes from substance-induced psychotic disorders (SIPD) to more severe illnesses, such as bipolar disorder or schizophrenia, occur frequently in young people aged 16–25 [11] and that no significant differences were found in the short-term prognoses of SIPD and non-substance-induced psychotic disorders (NSIPD) [11, 12].

Specialized early intervention services (EIS) are comprehensive outpatient treatments for patients with EP, where low-dose antipsychotic medications, cognitive–behavioral psychotherapy, family interventions, and motivational interviews are offered [13, 14]. A recent meta-analysis of randomized controlled trials (RCTs) of EIS reported that compared to treatment as usual, EIS were associated with significantly better outcomes in symptoms, functioning, remission, recovery, all-cause treatment discontinuation, and psychiatric hospitalization [3]. Specifically, the EIS groups had moderate-to-high within-group effect sizes for improvements in psychopathology and functioning: 57.3% achieved study-defined remission and 30.3% recovery [3]. The all-cause treatment discontinuation rates at 9–24-month follow-up were 9–15% [6–9], and the rehospitalization rates were 6–34% [15–21].

Although many individuals with EP are hospitalized during the first psychotic episode [22–24] and often experience their first hospitalization as traumatic [25], all EIS evaluated in recent RCTs commenced after hospitalization as outpatient and/or assertive outreach treatments, and do not include specialized inpatient treatment [3]. To our knowledge, only two studies of specialized inpatients treatments for EP exist. A 2-year follow-up pilot study examined inpatient CBT for EP and found lower relapse rates and lower recurrence times of psychotic symptoms compared to a supportive counselling group [26]. Another study that offered patients with EP outpatient, specialized inpatient and an intensive mobile team care found lower rehospitalization rates in comparison to patients not enrolled in the program [27]. Whereas both studies did examine inpatient treatment for EP, none of the studies offered a comprehensive specialized treatment program focused solely on the inpatient setting. Thus, little is known about the clinical development of patients with EP when enrolled in a specialized inpatient treatment program, as well as about the feasibility of such a program in inpatient settings.

### Aims of the study

The aim of study was to observe the development of clinical outcomes in young people with EP in a specialized inpatient treatment and assess the feasibility of such a specialized treatment in an inpatient setting.

In previous studies, positive attitudes towards psychiatric medication and patient satisfaction were associated with lower service disengagement, medication non-adherence, and involuntary admissions, and associated with improved psychopathology, functioning, and satisfaction with care [28–33]. Based on these findings, we assumed an increase in both primary outcomes (1) attitudes towards psychiatric medication, and (2) patient satisfaction of treatment after 6 weeks of treatment. To compare and validate our treatment with other EIS, we also analyzed the following secondary outcomes at a 6-week, 6-month and 12-month follow-up timepoint: symptom severity, functioning, and involvement in school or work, as well as all-cause treatment discontinuation, rehospitalizations, remission, and recovery.

## Materials and methods

### Study design

This was a prospective 1-year cohort study performed at “FRITZ” (early intervention and therapy center) in Berlin, Germany. The FRITZ intervention program was developed for young people with EP aged 18–35 years and offers in- and outpatient services [34, 35]. For the purpose of this study, only young people with EP who received inpatient treatment were approached.The FRITZ center is located in the inner city of Berlin, in a catchment area with a high proportion of young people (45.1%) [36], as well as high proportions of individuals with migration backgrounds, poverty, and alcohol and substance use [37].

### Study procedures

Recruitment for the study took place from December 2015 to May 2018. Individuals who fulfilled the inclusion criteria and were able to understand the nature and scope of the study were approached. Baseline assessments were carried out 24 h after written informed consent was obtained. Assessments took place at baseline, 6-week, 6-month and 12-month follow-up. After inpatient treatment, patients were discharged to the outpatient center of the hospital or to non-specialized psychiatrists and/or psychotherapists in private practice.

Inclusion criteria adhered to the EP definition [4] and were the following: (1) diagnosis of a psychotic disorder (ICD-10;World Health Organization [38], including substance-induced psychosis F1x.5, schizophrenia-spectrum-disorders F2x, mania with psychotic symptoms F30.2, bipolar affective disorder with psychotic symptoms F31.2/F31.5, and severe depression with psychotic symptoms F32.3/F33.3); (2) onset of the first psychotic episode did not exceed 5 years, or presentation to mental health services did not occur more than 5 years ago [4]; (3) willingness to participate in the therapy program at FRITZ; (4) sufficient intellectual abilities and proficiency in German to complete study procedures.

Sociodemographic and clinical data were assessed by independent researchers and FRITZ clinicians.

### Description of the intervention

The FRITZ intervention program was developed for young people with EP and followed international treatment guidelines [34, 35]. The program includes pharmacological, psychotherapeutic, and socio-therapeutic approaches. The individual and group cognitive behavioral therapies (CBT) were adapted for inpatient use from the Individual Resiliency Training by Penn, Meyer, Gottlieb, and colleagues [39]. Furthermore, metacognitive training by Moritz et al. [40] as well as vocational therapy implementing the principles of supported employment [41, 42] were offered. The family interventions consisted of individual sessions with patients and one or two family members, as well as of multifamily group interventions following the model provided by Glynn [43].

Out of the 31 essential components suggested by the First-Episode Psychosis Services Fidelity Scale (FEPS-FS) [44], most of the components were utilized and adapted to the inpatient setting. The adapted components implemented in the FRITZ treatment scored 88.39% in the total score of the FEPS-FS, compareable to 86% for the satisfactory programs [44].

Detailed descriptions of all interventions are provided in a manual [45]. The study staff was trained in multi-professional training workshops and was regularly supervised by K. Leopold and A. Bechdolf.

### Measurements

At baseline, a semi-structured interview based on ICD-10 was used to determine diagnosis [46]. Pathways to care was assessed by an adjusted version of the Pathways to Care Encounter Form [47]. Duration of untreated psychosis (in days) was assessed by an adapted version of the Nottingham Onset Schedule [48]. Furthermore, the Addiction Severity Index was utilized to assess substance use [49].

### Primary outcomes

The attitude towards psychiatric medications was assessed with the Drug attitude inventory (DAI-10), a self-report instrument assessing clinical dimensions relevant to non-adherence with good psychometric properties [50, 51]. Scoring ranges from −10 to + 10; a total score > 0 indicating a positive attitude towards psychiatric medications and a total score of < 0 indicating a negative attitude towards psychiatric medications.

Patient satisfaction with treatment was assessed through the Patient Satisfaction Questionnaire Short Form (PSQ-18) [49], a validated self-report instrument [52]. There are 18 statements which are ranked on a 5-point scale from “strongly agree” to “strongly disagree”. The questionnaire examines seven dimensions of patient satisfaction directed towards their psychiatrist/psychologist. The subscale *financial aspects* (consisting of two items) were excluded because these aspects are not relevant in the German mental health care system. The total score was calculated as the mean of all 16 items, with a higher score indicating more patient satisfaction in accordance with treatment [53].

### Secondary outcomes

Overall, illness severity was assessed using the Clinical Global Impression-Schizophrenia Scale (CGI-SCH Scale) [54], rated from 1 “absent” to 7 “extreme”. Psychotic symptom severity was assessed through the Positive and Negative Syndrome Scale (PANSS) [55]. The PANSS includes 30 items. Each item is rated on a scale from 1 “absent” to 7 “extreme”. The subscale scores of positive symptoms (7 items), negative symptoms (7 items), and general psychopathology (16 items) were computed by summing all respective item scores. The total PANSS score was computed by summing all subscales, with higher scores indicating higher symptom severity. Current level of functioning was assessed through the Global Assessment of Functioning (GAF) [56, 57] scale; with possible scores ranging from 100 “extremely high functioning” to 0 “severely impaired”.

All-cause treatment discontinuation was defined as not attending follow-up appointments assessed through medical records and patient interviews. Rehospitalization was defined as at least one psychiatric hospitalization with an overnight stay during the 12-month follow-up period and was assessed through electronic medical records.

Remission of illness was defined in accordance with Andreasen et al. [58] (PANSS items: P01, P02, P03, G05, G09, N01, N04, N06 ≤ 3, about  ≥6 month, at 6-month follow-up regardless the time criteria). Recovery was defined by remission criteria and GAF value $\ge$61 regardless the time criteria $\ge$2 years [59]. As this study was limited to 12 months, the time criterion for remission and recovery of 2 years was adapted to our timeframe.

### Other dimensions

The number of therapy sessions received throughout the FRITZ inpatient admissions was counted through the electronic medical records. The sessions encompassed individual vs. group therapy by psychiatrists, psychologists, other therapists (social workers, occupational therapists, physiotherapists), as well as individual and/or group family therapy sessions.

### Subgroup and dropout analyses

Due to the high percentage of patients with SIPD included in the study, a subgroup analysis was conducted to investigate the potential influence of different diagnostic entities on the illness trajectory when compared with the NSIPD group. The NSIPD group included the following diagnoses: schizophrenia, delusional disorder, acute and transient psychotic disorder, schizoaffective disorder, mania with psychotic symptoms, bipolar affective disorder, current episode manic with psychotic symptoms, and severe depressive episode with psychotic symptoms. The SIPD group entailed cannabis-induced disorder, other stimulants-induced disorder, and hallucinogen-induced disorder.

In addition, we investigated a dropout analyses to detect systematic differences of individuals who have not completed the survey.

### Statistical analysis

Power analyses were performed for both primary outcomes, in which a standard deviation twice the delta, an alpha error of 5%, and a power of 80% in a two-sided test was assumed each time. The analyses revealed that 51 patients were needed to detect a clinically meaningful improvement of 2 points in the DAI-10 at 6-week follow-up, whereas 73 patients were needed to show a clinically meaningful improvement of one point in the mean score of the PSQ-18 at week 6. Anticipating a dropout rate of 15%, a minimum of 85 patients had to be assigned to the trial [60]. No assumptions were made for any other measurements.

The differences in both primary outcomes between baseline and follow-ups were calculated with a repeated-measures ANOVA (Baseline, 6 weeks, 6 months, and 12 months). Significant main effects were further analyzed using paired *t* tests. Because three follow-up comparisons were included in the paired *t* tests, we adjusted the *p*-level to 0.017 (i.e., Bonferroni correction) [61]. Cohen´s *d* was used as the effect size measure and was computed using the standard deviations and correlations of the measured values. Effect sizes of *d* ≥ 0.2 are considered “small”, *d* ≥ 0.5 “medium”, and *d* ≥ 0.8 “large” [62].

Two independent methods to replace continuous missing data of primary and secondary outcomes (DAI-10, PSQ-18, CGI, PANSS, and GAF) were applied: last observation carried forward (LOCF) and multiple imputation (MI). The MI was calculated with five iterations and with an estimation-maximization algorithm. Imputed scores are defined as the range between the minimum and maximum of each scale. After data imputation, imputed and observed results were compared. The observed as well as imputed variables were normally distributed (*p* < 0.05, Greenhouse–Geisser correction). All statistical analyses were performed using SPSS version 22 for Windows [63].

## Results

### Sample characteristics

As the lost to follow-up rate at 6 months for primary outcome data was higher than expected (29.41% instead of 15.00%), 98 patients were enrolled in the study instead of 85. The enrolment procedure was the same for all recruited individuals. Whereas the PANSS positive score was less severe, the subsequently recruited individuals did not differ in comparison to the rest of the sample in terms of age, sex, years of education, duration of untreated psychosis (DUP), time between onset of illness and study enrolment, number of inpatient stays, rehospitalization at 6 and 12 months, and treatment discontinuation at 6 and 12 months (Supplement, Table S1). After enrolment, three patients withdrew their consent. Ultimately, the sample consisted of 95 inpatients with EP, of which 44 patients (46.4%) reported their first inpatient stay. The mean age of the sample was 26.17 years. The mean years of education (including college) were 14 years. 40% of the sample was unemployed. The majority of the patients (74.73%) received antipsychotic medication (mainly 2nd generation antipsychotics) at baseline. See Table 1 for patients´ sociodemographic and illness characteristics.

**Table 1:** Sociodemographic and clinical characteristics of the sample at baseline (*N* = 95)

Characteristics	*n*/*M*	SD/(%)
Demographics		
Age at admission	26.17	5.48
Female sex	36	(38.30)
Education (in years)	14.09	3.51
Occupational degree		
No degree	44	(46.30)
In education	9	(9.50)
University	25	(26.30)
Vocational education	11	(11.60)
University and vocational education	6	(6.30)
Occupation		
Unemployment	38	(40.40)
Employment	16	(17.00)
Pupil/student	29	(30.90)
Sick leave	11	(11.70)
Primary diagnoses		
F12.5 Cannabis-induced psychotic disorder	27	(28.40)
F1X.5 Other stimulants-induced psychotic disorder	16	(16.90)
F16.7 Hallucinogen-induced psychotic disorder	1	(1.00)
F20.X Schizophrenia	28	(29.50)
F22.0 Delusional disorder	1	(1.00)
F23.X Acute and transient psychotic disorder	9	(9.50)
F25.X Schizoaffective disorder	3	(3.20)
F30.2 Mania with psychotic disorder	1	(1.00)
F31.2 Bipolar affective disorder, current manic episode with psychotic symptoms	6	(6.30)
F32.3 Severe depressive episode with psychotic symptoms	3	(3.20)
Secondary diagnoses		
F1X.1 Substance abuse	18	(18.95)
F1X.2 Substance dependence	32	(33.68)
FX.X Other psychiatric diagnosis	5	(5.26)
DUP^a^, mean, median	99.97/28	202.93
Time to study enrolment^b^, mean, median	410.44/250.50	468.37
Positive psychiatric family history^c^	56	(58.90)
Number of inpatient stays at admission^d^	2.16	1.8

*M* mean; *SD* standard deviation

^a^*DUP* duration of untreated psychosis in days, *n* = 17 missing (NOS, adapted version [48])

^b^Time between onset of first-episode psychosis and study enrolment

^c^Positive psychiatric family history, *n* = 2 missing

^d^Number of inpatient stays at index admission includes the first inpatient stay at FRITZ

### Feasibility of multi-professional specialized inpatient treatment and treatment use

Eighty-eight patients (92.63%) received inpatient treatment for 6 weeks or longer. The mean duration of days in inpatient treatment was 31.28 days (SD = 20.48; median = 27). The mean number of individual CBT sessions was 7.37 (SD = 5.19; median = 6) and group CBT sessions (including psychoeducational interventions and substance use treatment) was 8.71 (SD = 6.39; median = 8). The mean number of other non-CBT individual therapy sessions was 2.35 (SD = 4.61; median = 1) and that of other group therapies was 7.56 (SD = 6.83; median = 6). Family members of 65 patients (67.70%) took part in patients’ individual therapy sessions. The families of 21 (21.10%) patients attended family group interventions focusing on psychoeducation, problem solving, and crisis management.

### Outcomes

All primary and secondary outcomes showed significant changes over time. The significant main effects of the repeated-measures ANOVA (Supplement Table S2) were further analyzed in the paired *t *tests.

### Acute-treatment results at 6-week follow-up

The primary outcomes, attitude towards medications, and patient satisfaction with treatment changed significantly from baseline to week 6 (Table 2). Significant changes were found for the secondary outcomes CGI, PANSS positive, PANSS general, PANSS total, and GAF (Table 2). No significant change was found for the PANSS negative score. No differences in the pattern of results for all measures were found when comparing the observed, the LOCF, and the MI data (Supplement, Table S3).

**Table 2:** Clinical symptoms, functioning, and psychosocial concepts at baseline and follow-ups: paired *t* tests of observed data

Outcome	Baseline vs. 6-week follow-up						Baseline vs. 6-month follow-up						Baseline vs. 12-month follow-up					
	*n*	*M*	SD	Δ^change^	*p*	*d *(95% CI)	*n*	*M*	SD	Δ^change^	*p*	*d* (95% CI)	*n*	*M*	*SD*	Δ^change^	*p*	*d *(95% CI)
DAI-10^a^	48	1.25 4.25	4.49 3.39	3.00	< 0.001	0.55 (0.15–0.96)	40	0.75 2.90	4.85 4.62	2.15	<0.05	0.35 (−0.09–0.79)	33	0.61 3.64	5.01 4.05	3.03	< 0.01	0.52 (0.02–1.01)
PSQ-18^b^	59	3.52 3.83	0.64 0.44	0.21	< 0.01	0.40 (0.04–0.77)	53	3.52 3.84	0.55 0.48	0.32	< 0.01	0.43 (0.04–0.81)	43	3.61 3.74	0.58 0.56	0.13	0.27	0.17 (0.26–0.59)
CGI^c^ global	65	4.34 2.85	1.20 1.23	1.49	< 0.001	−1.06 (−1.42 to [−0.69])	60	4.23 2.62	1.24 1.21	1.61	< 0.001	−0.98 (−1.36 to [−0.6])	55	4.24 2.60	1.25 1.33	1.64	< 0.001	−1.12 (−1.52 to [−0.72])
PANSS^d^ positive	66	16.82 10.35	6.50 3.50	6.47	< 0.001	−0.80 (−1.16 to [−0.44])	59	16.84 9.57	7.01 3.64	7.27	<0.001	−0.81 (−1.19 to [−0.43])	55	16.67 9.56	6.68 3.95	7.11	< 0.001	−0.79 (−1.18 to [−0.41])
PANSS^d^ negative	64	14.23 12.89	7.01 6.38	1.34	0.10	−0.20 (−0.55 to [−0.15])	59	13.73 11.51	6.86 5.06	2.22	< 0.05	−0.28 (−0.65 to [−0.08])	55	14.05 11.11	7.21 4.79	2.94	< 0.01	−0.38 (−0.76 to [−0.01])
PANSS^d^ general	65	32.71 25.88	9.59 7.82	6.83	< 0.001	−0.66 (−1.01 to [−0.30])	59	32.17 23.02	9.90 5.91	9.15	<0.001	−0.69 (−1.06 to [−0.32])	55	32.53 22.47	10.02 6.01	10.06	<0.001	−0.73 (−1.12 to [−0.35])
PANSS^d^ total	64	64.17 49.22	18.13 15.73	14.95	< 0.001	−0.77 (−1.13 to [−0.41])	59	62.75 44.10	18.82 12.54	18.65	< 0.001	−0.78 (−1.16 to [−0.41])	55	63.25 43.15	19.04 12.18	20.10	< 0.001	−0.76 (−1.15 to [−0.38])
GAF^e^	64	46.89 60.72	13.51 14.47	13.83	< 0.001	0.97 (0.6–1.33)	60	47.10 66.07	13.37 16.39	18.97	<0.001	1.21 (0.82–1.60)	53	48.28 67.81	13.67 17.31	19.58	<0.001	1.25 (0.84–1.67)

*M*  mean; *SD* standard deviation; Δ^change^  change in an outcome between assessment time points; *p* = 0.02, significance Level; *d*  effect size;* CI* critical interval

^a^DAI-10, Drug Attitudes Inventory [50, 51]

^b^PSQ-18, Patient Satisfaction Questionnaire [52]

^c^CGI global, Clinical Global Impression-Schizophrenia Scale global value [54]

^d^PANSS (positive, negative, general, total), Positive and Negative Syndrome Scale with subscales positive and negative symptoms, general symptoms and total score [55]

^e^GAF, Global Assessment of Functioning Scale [56, 57]

### Results at 6-month and 12-month follow-up

The change in attitude towards medication found at 6 weeks was maintained at the 12-month follow-up mark. Patient satisfaction with treatment increased significantly between baseline and 6-month follow-up (*p* < 0.01; Table 2). There was no significant increase in patient satisfaction at 12 months (*p* = 0.27; Table 2). These findings were replicated by the LOCF and MI data analyses (Supplement Tables S3/S4).

The significant increases in the secondary outcomes (CGI, PANSS positive, PANSS general, PANSS total, and GAF) found at 6 weeks were maintained at 6 months and at 12 months. The significant change in the PANSS negative score remained small over a 12-month period. Again, almost all results could be replicated with LOCF and the MI data.

Remission was achieved by 71.67% (*N* = 43) of the participants at 6 months and 66.04% (*N* = 35) at 12 months. Recovery was achieved by 55.00% (*N* = 33) of the participants at 6 months and 52.83% (*N* = 28) at 12 months. The all-cause treatment discontinuation rate was 13.69% (*N* = 13) at 6 months and 35.79% (*N* = 34) at 12 months. The rehospitalization rate was 30.53% (*N* = 29) at 6 months and 43.16% (*N* = 41) at 12 months. The mean number of hospitalizations was 0.38 (SD = 0.62; *r* = 0–2) at 6 months and 0.66 (SD = 0.88; *r* = 0–3) at 12 months. Involvement in school or work increased from 47.9% at baseline to 58.2% at 12 months (χ^2^(3) = 9.50, *p* = 0.02, *n* = 46).

### Subgroup analyses between non substance-induced psychotic disorders vs. substance-induced psychotic disorders

The subgroups did not differ in terms of age, sex, years of education, DUP, time between illness onset and study enrolment, number of inpatient stays, diagnoses, rehospitalization at 6 and 12 months, or treatment discontinuation at 6 and 12 months (Table 3). At baseline, the PANSS positive score was significantly higher in the SIPD group (*t*(93) = −3.33, *p* = 0.001), while all other subscales of the PANSS did not differ at baseline.

**Table 3:** Sociodemographic and clinical characteristics by subgroups: NSIPD vs. SIPD

	NSIPD *M*, SD/*n*	*n*	SIPD *M*, SD/*n*	*n*	Significance levels
Age	26.25 ± 5.47	51	26.07 ± 5.56	44	*t*(93) = 0.16, *p* = 0.87
Female gender	22	51	14	44	χ^2^(1) = 1.29, *p* = 0.26
Education (years)	13.89 ± 3.57	51	14.26 ± 3.46	43	*t*(92) = −0.49, *p* = 0.62
DUP^a^ (days), median	28	39	28	39	*U* = 759, *z* = −0.01, *p* = 0.99
Time to enrolment^b^ (days), median	292	39	164	39	*U* = 585, *z* = −1.75, *p* = 0.08
Number of inpatient stays	2.41 ± 1.75	51	1.86 ± 1.77	44	*t*(93) = 1.52, *p* = 0.13
DAI^c^ baseline	1.06 ± 4.32	34	0.14 ± 5.22	28	*t*(60) = 0.76, *p* = 0.45
PSQ^d^ baseline	3.46 ± 0.59	44	3.61 ± 0.57	37	*t*(79) = −1.15, *p* = 0.25
PANSS^e^ baseline					
Postive	15.74 ± 6.15	51	20.14 ± 6.67	44	*t*(93) = −3.34, *p* = 0.001
Negative	14.77 ± 7.09	50	12.64 ± 5.84	44	*t*(92) = 1.53, *p* = 0.13
General	31.72 ± 8.24	50	32.09 ± 9.80	43	*t*(91) = −0.20, *p* = 0.84
Total	61.25 ± 17.99	50	64.14 ± 19.55	43	*t*(91) = −0.75, *p* = 0.46
GAF^f^ baseline	46.86 ± 13.91	51	45.86 ± 13.35	44	*t*(93) = 0.36, *p* = 0.72
Rehospitalization					
6-month (yes)	15	51	14	44	χ^2^ (1) = 0.06 *p* = 0.80
12-month (yes)	22	51	19	44	χ^2^ (1) = 0.00 *p* = 0.99
All-cause treatment discontinuation					
6-month (yes)	2	48	3	41	χ^2^(1) = 0.41 *p* = 0.52
12-month (yes)	4	43	9	31	χ^2^(1) = 4.84, *p* = 0.03
Remission					
6-month (yes)	27	36	16	20	χ^2^(1) = 0.18, p = 0.67
12-month (yes)	22	29	13	18	χ^2^(1) = 0.18, *p* = 0.67
Recovery					
6-month (yes)	21	36	12	20	χ^2^(1) = 0.02, *p* = 0.90
12-month (yes)	18	29	10	18	χ^2^(1) = 0.20, *p* = 0.66
Involvement in school/work					
6-month (yes)	22	37	17	22	χ^2^(1) = 1.95, *p* = 0.16
12-month (yes)	18	32	14	23	χ^2^(1) = 0.12, *p* = 0.73

*M* mean; *SD* standard deviation; *p* = 0.05, significance level; *NSIPD *  non substance-used psychotic disorder; *SIPD* substance-induced psychotic disorder

^a^DUP, duration of untreated psychosis in days, *n* = 17 missing (NOS, adapted version [48])

^b^Time between onset of first-episode psychosis and study enrolment

^c^DAI-10, Drug Attitudes Inventory [50, 51]

^d^PSQ-18, Patient Satisfaction Questionnaire [52]

^e^PANSS at Baseline (positive, negative, general, total), Positive and Negative Syndrome Scale with subscales positive and negative symptoms, general symptoms and total score [55]

^f^GAF, Global Assessment of Functioning Scale [56, 57]

The NSIPD group showed similar results to the results from the total study sample in most measures (Supplement, Table S5). There were significant increases in both attitude towards medication (*p* < 0.01, Supplement, Table S6) and patient satisfaction with treatment (*p* < 0.01, Supplement, Table S5) between baseline and 6 weeks of treatment. Whereas there continued to be a significant increase in attitude towards medication up until the 12-month follow-up mark (*p* < 0.01, Supplement, Table S5), no significant changes in patient satisfaction were found after the 6-week timepoint. Significant changes were also found for the secondary outcomes CGI, PANSS positive, PANSS general, PANSS total, and GAF at all assessment timepoints (See Supplement, Table S5). No significant change was found for the PANSS negative score between baseline and the 6-week timepoint, yet, a significant change was found at 12 months (*p* = 0.01, Supplement, Table S5). No significant change for involvement in school or work was found at 12 months for the NSIPD group (χ^2^ (3) = 6.0, *p* = 0.11, *n* = 28).

The SIPD group showed a significant increase in attitude towards medications after 6 weeks (*p* = 0.02, Supplement, Table S6); however, no significant changes were found at 6 months or 12 months. No significant changes were found for patient satisfaction at any study assessment timepoints (Supplement, Table S6). Furthermore, significant changes were found for the secondary outcomes CGI, PANSS positive, PANSS general, PANSS total, and GAF at all assessment timepoints (Supplement, Table S6). No significant change was found for PANSS negative score at any study assessment timepoints. No significant change for involvement in school or work was found at 12 months for the SIPD group (χ^2^(3) = 4.11, *p* = 0.25, *n* = 18).

### Dropout analyses

The analyses showed that the dropouts did not differ in terms of age, sex, DUP, time between illness onset and study enrolment, number of inpatient stays, diagnoses (SIPD versus NSIPD), rehospitalization at 6 months and 12 months, and treatment discontinuation at 6 months (Table 4). The dropouts showed significant fewer years of education, a higher all-cause treatment discontinuation rate at 12 months, and a higher PANSS positive score at baseline.

**Table 4:** Dropout analyses sociodemographic and clinical characterstics

	Non-dropouts *M*, SD/*n*	*n*	Dropouts *M*, SD/*n*	*n*	Significance levels
Age	26.87 ± 5.32	47	25.47 ± 5.62	48	*t*(93) = 1.25, *p* = 0.22
Female sex	22	47	14	48	χ^2^(1) = 3.14, *p* = 0.08
Education (years)	15.25 ± 3.55	46	12.92 ± 3.09	48	*t*(92) = 3.40, *p* = 0.01
DUP^a^ (days), median	21	40	30	38	*U* = 710, *z* = −0.49, *p* = 0.62
Time to enrolment^b^ (days), median	250,5	40	230	38	*U* = 732, *z* = −0.28, *p* = 0.78
Number of inpatient stays	2.09 ± 1.65	47	2.23 ± 1.89	48	*t*(93) = −0.40, *p* = 0.69
Ratio between NSIPD vs. SIPD^c^	29/18	47	22/26	48	χ^2^(1) = 2.41, *p* = 0.12
DAI^d^ baseline	0.63 ± 4.81	38	0.67 ± 4.71	24	*t*(60) = −0.028, *p* = 0.97
PSQ^e^ baseline	3.56 ± 0.62	44	3.49 ± 0.53	37	*t*(79) = 0.56, *p* = 0.58
PANSS^f^ baseline					
Postive	16.38 ± 6.94	47	19.15 ± 6.30	48	*t*(93) = −2.03, *p* = 0.05
Negative	14.19 ± 7.28	47	13.28 ± 5.86	47	*t*(92) = 0.67, *p* = 0.50
General	32.57 ± 10.72	47	31.20 ± 6.73	46	*U* = 1096.50, *z* = −0.88, *p* = 0.93
Total	63.15 ± 20.18	47	62.04 ± 17.30	46	*t*(91) = 0.29, *p* = 0.77
GAF^g^ baseline	48.72 ± 13.48	47	44.13 ± 13.44	48	*t*(93) = 1.66, *p* = 0.09
Rehospitalization					
6-month (yes)	12	47	17	48	χ^2^(1) = 1.09, *p* = 0.30
12-month (yes)	18	47	23	48	χ^2^(1) = 0.90, *p* = 0.34
All-cause treatment discontinuation					
6-month (yes)	2	47	3	42	χ^2^(1) = 0.35, *p* = 0.56
12-month (yes)	4	47	9	27	χ^2^(1) = 7.30, *p* = 0.01
Involvement in school/work					
6-month (yes)	32	46	7	13	χ^2^(1) = 1.12, *p* = 0.29
12-month (yes)	28	47	4	8	χ^2^(1) = 0.26, *p* = 0.61

*M*  mean; *SD*  standard deviation; *p* = 0.05, Significance level

^a^*DUP* duration of untreated psychosis in days, *n* = 17 missing (NOS, adapted version [48])

^b^Time between onset of first-episode psychosis and study enrolment

^c^Ratio between NSIPD vs. SIPD in the subgroups Non-droupouts and droupouts

^d^*DAI-10* Drug Attitudes Inventory [50, 51]

^e^*PSQ-18* Patient Satisfaction Questionnaire [52]

^f^PANSS at Baseline (positive, negative, general, total), Positive and Negative Syndrome Scale with subscales positive and negative symptoms, general symptoms and total score [55]

^g^GAF at Baseline, Global Assessment of Functioning Scale [56, 57]

## Discussion

This study presents findings about the development of clinical outcomes of young people with EP in a specialized inpatient treatment over a 12-month period, as well as about the feasibility of such an intervention in an inpatient setting.

The FRITZ model adapted and implemented international guidelines for outpatient first-episode psychosis services to an inpatient setting. With the vast majority of the sample accepting 6 weeks of inpatient treatment, the FRITZ model could be described as well accepted and feasible.

In accordance with our assumptions, there was a significant increase in attitude towards medication and patient satisfaction with treatment after a 6-week inpatient treatment program. The significant increase of attitudes towards medication was also present at 12-month follow-up and the significant increase of patient satisfaction with treatment was maintained at 6-month follow-up. Other clinical outcomes (CGI, PANSS, and GAF) also increased significantly after 6-week inpatient treatment and were maintained over the 12-month follow-up, except for the PANSS negative score which increased significantly at 12 months.

At baseline, patients displayed a positive attitude towards medication, which increased throughout a 12-month period. The increase in attitude towards medication in this study was slightly higher than results presented at 12 months in a naturalistic treatment study of patients with schizophrenia [64]. One reason for this could be the consistent use of lower second-generation antipsychotic doses, which may have resulted in fewer medication side effects. A factor likely to influence a more positive attitude towards medication is the reduction and/or prevention of medication side effects. Studies in EP indicate a negative attitude towards medication as an early predictor for medication non-adherence or discontinuation [28–33, 65–67]. Thus, the more positive the attitude towards medication, the more likely patients will be medication adherent throughout long-term treatment. Yet, the all-cause treatment discontinuation and rehospitalization rates suggest that there are other factors, which may influence a positive attitude towards medication and, thus, elicits better medication adherence (e.g., a reliable and stable therapeutic relationship). It is therefore, vital to critically consider how treatment continuity and adherence can be maintained, especially for patients with a high risk of all-cause treatment discontinuation (e.g., case management).

High baseline scores and significant changes throughout the follow-up period were also found for patient satisfaction, which supports the notion that specialized inpatient treatment for patients with EP seems to be well perceived (at least until month 6) and could be beneficial for the short-term outcome. This change in patient satisfaction could be related to changes in other clinical outcomes (e.g., PANSS and GAF) as found in other studies [31, 32]. Other research assessing EP treatments found that high levels of patient satisfaction were linked to treatments that allowed for a high degree of individualization and shared decision-making [68].

Compared with the EIS samples included in the meta-analysis by Correll and colleagues [3], the FRITZ sample showed similar results in terms of some demographic (age: 23–29 years, sex: > male, first inpatient admission: 86–100%) and clinical (GAF: 36.22–50.00, PANSS positive score: 12.32–20.75) characteristics. In contrast to the EIS samples, the FRITZ sample showed lower PANSS negative scores (EIS: 16.99–23.7), lower DUP (EIS: 178–300 days), as well as a higher diagnoses’ heterogeneity (higher proportion of substance-induced psychosis), which may reduce the comparability of the samples.

Both the EIS sample and the FRITZ sample showed decreases in positive psychotic symptoms and general symptoms with medium-to-large-effect sizes in the respective investigation period (EIS: PANSS positive: 5.8 [21], 7,52 [18] and 10.7 [19]; PANSS general: 8.0 [21], 3.76 [18], 23.3 [19]; GAF: 2.4 [21], 22.22 [18], 23.8 [19]). The same applies to the decrease of negative symptoms with small-effect sizes, which are also comparable to the EIS trials (PANSS negative 3.2 [21], 1.36 [18] and 12.7 [19]. Likewise, the rates of remission of 66.04% and recovery of 52.83% at 12 months in the FRITZ sample appear to be similar to the 57.30% remission rate and higher than the 30.30% recovery rate in the EIS trial meta-analyses [3]. However, recovery rates in this study disregarded the 2-year time criterion [59], because the overall duration of follow-up was limited to 12 months. Thus, this may be a reason for the higher rate found in our study compared to the EIS trials.

The all-cause treatment discontinuation rate at 6 months in the FRITZ sample (13.69%) appear similar to the sample of the EIS trials (9–15%) [15–19]. However, there was a substantial increase in the all-cause treatment discontinuation rate at 12 months in the FRITZ sample (35.79%) compared to EIS sample (9–15%) [15–19]. Similar patterns of results were found for rehospitalization rates, with the FRITZ sample obtaining similar rates at 6 months (30.53%), but slightly higher rates at 12 months (43.16%) compared to the EIS sample (at 9–24 months: 6–34%) [3].

The higher treatment discontinuation and rehospitalization rates at 12 months in the FRITZ sample could have many explanations. On one hand, it could be explained by the less intense outpatient treatment following the inpatient stay as compared to the EIS. In contrast to the EIS treatments, assertive outreach could not be offered within the German health care system, if patients were not able or not willing to attend outpatient appointments. Thus, this may be a reason for higher treatment discontinuations and subsequently lead to more relapses and more frequent rehospitalizations. On the other hand, as the FRITZ study reported a high acceptance of the inpatient treatment, some patients may have preferred inpatient stays to outpatient treatment, which may also have to lead to a higher rate of rehospitalization.

Since the FRITZ sample included a higher proportion of SIPD than other cohorts [3], we conducted a subgroup analyses of the SIPD and the NSIPD group. In summary, the differences between the SIPD and the NSPD compared to the overall sample were small. Compared to the overall result of the study sample, the NSIPD group only differed in terms of finding no significant changes in patient satisfaction after 6 weeks. The SIPD group showed a significant increase in attitude towards medication after 6 weeks of inpatient therapy, followed by no significant changes in attitude throughout the follow-up period. Furthermore, there was no significant change regarding treatment satisfaction. The results indicate that the SIPD group appears to be less responsive and more critical towards therapy. A potential explanation is that patients may lack insight into or deny their substance misuse/dependency [69–71]. Results showed a higher rate of all-cause treatment discontinuation for the SIPD group (at 12 months), but not for rehospitalization rates. The difference in all-cause treatment discontinuation rates can be explained through either a lack of insight into substance misuse/dependency leading to resistance towards and rejection of therapy, or due to cravings [69].

In summary, the results of both groups did not reveal a clear distinction between the SIPD and NSIPD groups in terms of psychotic symptom severity, remission and relapse rates, and vocational status [11, 12]. As no significant differences were found in the sociodemographic or clinical characteristic between the groups, our findings are align and support other research findings that EP should include an array of different psychotic diagnostic entities [11, 12]. As there are high conversion rates from SIPD to bipolar and schizophrenia-spectrum-disorders [11] and little is known about potential markers to differentiate between nonconverters and converters, including SIPD in EP research may help us gain a more comprehensive understanding of the illness. This in turn allows for earlier implementations of specific interventions for converters to try and prevent the development of severe illness trajectories.

The results of the present study need to be interpreted within its limitations. First, the absence of a control group did not allow for any causal interpretations regarding the observed changes to be made and cannot be solely attributed to the specialized treatment per se. However, as our results closely resembled the findings reported from EIS treatments (over 12 month), it is unlikely that similar results could stem from treatments implemented in standard clinical care.

Another limitation concerns the relatively small sample size. This may have led to an overestimation of the changes seen in our clinical measures. EIS studies with more participants [16] showed lower effect sizes than those with fewer participants [19].

Furthermore, the varying number of participants in the different clinical scales, and the high dropout rate after 12 months (50.5%) should also be regarded as limitations. The restrictive handling of the data in the repeated-measures ANOVA showed changes over time. Most of the results were replicated in smaller analysis units (paired t tests) and in data replacement procedures; thus, it could be assumed that the results were stable. The data replacement and estimation also tried to deal with dropouts. The dropout analysis revealed that patients who have fewer years of education and more serve symptoms had a higher risk of discontinuing treatment.

Another limitation is the diagnostic heterogeneity of the present sample. However, over the past few years, research within the EP field has broadened its definition of psychosis to include a variety of different psychotic disorders. Yet, the existing, although very limited findings suggest that diagnoses are prone to change over the course of an illness [9, 10] and early prognoses between NSIPD and SIPD hardly differ [11, 12].

In addition, PANSS and GAF measures may not fully evaluate the symptomatology or general functioning of SIPD or affective psychosis, which therefore may reduce the validity of the respective psychopathology and recovery findings. Yet, to our knowledge, no specific scales for SIPD or affective psychosis have been developed, which is why already validated and well-established scales were used in this study.

Furthermore, as our sample had a relatively low duration of untreated psychosis compared to other naturalistic EP studies, it might reduce the ability to compare our samples with others. However, it could also mean that patients included in this study had earlier access to specialized interventions, which is thought to prove more successful in treating psychotic symptoms [5]. Another potential reason as to why duration of untreated psychosis was lower might be due to the heterogeneity of the variables onset of the psychosis within last 5 years and the time between the onset of the psychosis to inclusion in the study. However, these variables represent a realistic representation of everyday clinical life.

Of note is that some of the clinical interviewers were involved in patients’ treatment, which may have led to biased rating of psychopathology [72]. However, the findings in the psychopathology ratings were in line with several other outcomes which are less likely to be biased, e.g., the number of rehospitalizations or the self-reported measures like attitudes towards medication and patient satisfaction. Thus, this limitation is unlikely to have substantially influenced the results. And lastly, this was a 1-year study and longer term outcomes need to be investigated further.

Taken together, the results show a positive development in many clinical variables in young people with EP treated at the FRITZ specialized inpatient ward. However, the clinical development also shows that additional interventions need to be implemented within this specialized inpatient treatment to reduce treatment discontinuation and rehospitalization. As higher treatment discontinuation rates were specifically linked to SIPD, fewer years of education, and more severe positive symptoms, it is important to take particular note of these factors in clinical practice and to try to build a strong therapeutic alliance with the patients, as well as identify barriers for treatment adherence. Additionally, more specialized CBT or social skills training need to be added to improve early recovery from negative symptoms [73]. Nevertheless, comprehensive EIS and outpatient treatments seem essential to improve patient´s engagement throughout long-term treatment [27]. However, since many young people with EP are hospitalized during their first psychotic episodes [22–24], specialized inpatient treatment for this population may represent the first step of specialized targeted interventions, and widen the scope of current well-evaluated and effective EIS. Thus, combining specialized inpatient treatment with outpatient EIS in young people with EP may help reduce and alleviate potential devastating consequences of EP for individuals, families, and societies. However, the findings of our study need to be replicated in controlled trials.

## Supplementary Information

Below is the link to the electronic supplementary material.

Supplementary file1 (DOCX 58 KB)

## Data Availability

Raw data were generated at Department of Psychiatry, Psychotherapy and Psychosomatic Medicine, Vivantes Hospital Am Urban, Berlin. Data are available from the corresponding author upon reasonable request.
