# Supplementary material for: Specialized inpatient treatment for young people with early psychosis: acute-treatment and 12-month results
Source: Eur Arch Psychiatry Clin Neurosci. 2022 Feb 9;272(7):1–14. doi: 10.1007/s00406-022-01379-8 (PMC9508217; doi:10.1007/s00406-022-01379-8)
Supplement: Supplementary file 1 — Supplementary file1 (DOCX 58 KB) [file 406_2022_1379_MOESM1_ESM.docx]

**Table S1.** Sociodemographic and clinical characteristics of subsequently recruited individuals (*n*=13)

| Characteristics | *N* = 13  *n/*M | SD*/(%)* |
| --- | --- | --- |
| **Demographics**  Age at admission  Female sex  Education (in years)  **Occupational degree**  No degree  In education  University  **Occupation**  Unemployment  Employment  Pupil/student  Sick leave | 27.47  7  15.27  7  1  5  7  1  4  1 | 5.83  (53.80)  3.77  (53.80)  (7.70)  (38.50)  (53.80)  (7.70)  (30.80)  (7.70) |
| **Primary diagnoses**  F12.5 Cannabis-induced psychotic disorder  F1X.5 Other stimulants-induced psychotic disorder  F16.7 Hallucinogen-induced psychotic disorder  F20.X Schizophrenia  F22.0 Delusional disorder  F23.X Acute and transient psychotic disorder  F25.X Schizoaffective disorder  **Secondary diagnoses**  F1X.2 Substance dependence  DUP**^a^**, mean, median  Time to enrolment**^b^**, mean, median  Positive psychiatric family history**^c^**  Number of inpatient stays at admission**^d^** | 1  1  1  6  1  2  1  3  59.78/ 28  486.33/ 317.00  7  3.0 | (7.70)  (7.70)  (7.70)  (46.15)  (7.70)  (15.40)  (7.00)  (23.10)  70.10  379.76  (53.80)  2.94 |

*M*=mean; *SD*=standard deviation.

**^a^**DUP, duration of untreated psychosis in days, *n* = 4 missing (NOS, adapted version [48]).

**^b^**Time between onset of first-episode psychosis and study enrolment.

**^c^**Positive psychiatric family history.

**^d^**Number of inpatient stays at index admission includes the first inpatient stay at FRITZ.

**Table S2.** Clinical symptoms, functioning and psychosocial concepts at baseline and follow-ups: repeated measures ANOVA of observed and replaced data

|  | Observed data | | | LOCF data | | | MI data | | |
| --- | --- | --- | --- | --- | --- | --- | --- | --- | --- |
| Outcome | *n* | *M (SD)* | *F* | *n* | *M (SD)* | *F* | *n* | *M (SD)* | *F* |
| **DAI-10^a^**  Baseline  6-weeks  6-months  12-months | 28  28  28  28 | 1.29 (4.63)  4.93 (3.15)  3.36 (4.32)  4.50 (3.47) | F_(2.04, 55.12)_=7.62, *p*<.01 | 62  62  62  62 | 0.65 (4.73)  2.97 (4.49)  2.16 (4.66)  2.65 (4.52) | F_(1.97,121.14)_=6.94, *p*=.001 | 95  95  95  95 | 0.75 (4.10)  4.13 (3.14)  2.82 (3.70)  4.11 (3.07) | F_(2.64, 248.46)_=23.95, *p*<.001 |
| **PSQ-18^b^**  Baseline  6-weeks  6-months  12-months | 36  36  36  36 | 3.57 (0.60)  3.87 (0.41)  3.91 (0.48)  3.82 (0.54) | F_(1.79, 62.61)_=4.80, *p*<.05 | 81  81  81  81 | 3.53 (0.58)  3.76 (0.44)  3.77 (0.48)  3.69 (0.51) | F_(1.94, 154.81)_= 7.6, *p*=.001 | 95  95  95  95 | 3.53 (0.61)  3.78 (0.45)  3.79 (0.53)  3.77 (0.65) | F_(2.76, 259.69)_= 6.23, *p*<.01 |
| **CGI^c^ _global_**  Baseline  6-weeks  6-months  12-months | 46  46  46  46 | 4.26 (1.32)  2.63 (1.25)  2.39 (1.18)  2.50 (1.24) | F_(2.51, 112.91)_=35.13, *p*<.001 | 95  95  95  95 | 4.26 (1.22)  3.24 (1.37)  3.06 (1.37)  3.08 (1.41) | F_(2.27, 213.04)_= 41.01, *p*<.001 | 95  95  95  95 | 4.26 (1.22)  2.83 (1.19)  2.57 (1.19)  2.72 (1.21) | F_(2.76, 259.43)_=54.28, *p*<.001 |
| **PANSS^d^ _positive_**  Baseline  6-weeks  6-months  12-months | 47  47  47  47 | 16.38 (6.94)  9.45 (2.95)  8.87 (3.08)  8.89 (2.99) | F_(1.60, 73.46)_=38.40, *p*<.001 | 95  95  95  95 | 17.78 (6.73)  13.28 (6.50)  12.43 (6.35)  12.35 (6.25) | F_(1.71, 160.36)_= 45.24, *p*<.001 | 95  95  95  95 | 17.78 (6.73)  10.31 (3.01)  9.55 (3.05)  9.72 (3.16) | F_(1.85, 173.62)_=92.13, *p*<.001 |
| **PANSS^d^ _negative_**  Baseline  6-weeks  6-months  12-months | 45  45  45  45 | 14.31 (7.38)  11.98 (6.10)  11.76 (5.58)  11.00 (4.79) | F_(2.31, 101.42)_=4.54, *p*<.05 | 94  94  94  94 | 13.73 (6.58)  12.81 (6.09)  12.39 (5.70)  12.01 (5.38) | F_(2.30, 213.70)_= 3.82, *p*<.05 | 95  95  95  95 | 13.69 (6.56)  12.88 (5.47)  11.42 (4.14)  11.09 (3.86) | F_(2.51, 236.27)_=8.46, *p*<.001 |
| **PANSS^d^ _general_**  Baseline  6-weeks  6-months  12-months | 46  46  46  46 | 32.78 (10.74)  24.74 (7.51)  22.54 (6.33)  21.54 (5.10) | F_(2.17, 97.52)_=24.48, *p*<.001 | 93  93  93  93 | 31.89 (8.95)  27.12 (7.79)  25.41 (7.55)  25.03 (7.54) | F_(1.95, 179.56)_= 28.49, *p*<.001 | 95  95  95  95 | 31.88 (8.86)  26.03 (6.65)  23.12 (4.88)  22.60 (4.79) | F_(2.32, 218.24)_=48.95, *p*<.001 |
| **PANSS^d^ _total_**  Baseline  6-weeks  6-months  12-months | 45  45  45  45 | 64.04 (20.15)  46.24 (14.61)  43.24 (13.52)  41.64 (10.49) | F_(2.04, 89.91)_=28.73, *p*<.001 | 95  95  95  95 | 62.59 (18.68)  52.03 (18.33)  49.57 (17.45)  48.74 (17.10) | F_(1.90, 178.50)_= 33.02, *p*<.001 | 95  95  95  95 | 62.59 (18.68)  48.13 (13.77)  44.04 (10.14)  43.21 (9.57) | F_(2.16, 202.58)_=58.87, *p*<.001 |
| **GAF^e^**  Baseline  6-weeks  6-months  12-months | 43  43  43  43 | 48.86 (14.04)  62.72 (14.28)  68.93 (17.11)  68.07 (17.03) | F_(2.35, 98.70)_=28.12, *p*<.001 | 95  95  95  95 | 46.40 (13.59)  55.72 (15.95)  60.23 (17.75)  60.76 (18.08) | F_(2.28, 214.21)_= 42.39, *p*<.001 | 95  95  95  95 | 46.40 (13.59)  60.71 (12.02)  65.76 (13.30)  67.87 (13.26) | F_(2.67, 251.30)_= 82.31, *p*<.001 |

LOCF= Last observation carried forward; MI= Multiple imputation; *M*=mean; *SD*=standard deviation; *p*=.02, significance Level.

**^a^**DAI-10, Drug Attitudes Inventory [50, 51].

**^b^**PSQ-18, Patient Satisfaction Questionnaire [52].

**^c^**CGI global, Clinical Global Impression-Schizophrenia Scale global value [54].

**^d^**PANSS (positive, negative, general, total), Positive and Negative Syndrome Scale with subscales positive and negative symptoms, general symptoms and total score [55].

**^e^**GAF, Global Assessment of Functioning Scale [56, 57].

**Table S3.** Clinical symptoms, functioning and psychosocial concepts at baseline and follow-ups: paired t-tests of LOCF data

|  | Baseline vs. 6-week follow-up | | | | | Baseline vs. 6-month follow-up | | | | | Baseline vs. 12-month follow-up | | | | |
| --- | --- | --- | --- | --- | --- | --- | --- | --- | --- | --- | --- | --- | --- | --- | --- |
| Outcome | *n* | *M* | *SD* | *p* | *d*  (95% CI) | *n* | *M* | *SD* | *p* | *d*  (95% CI) | *n* | *M* | *SD* | *p* | *d*  (95% CI) |
| DAI-10**^a^** | 62 | 0.65  2.97 | 4.73  4.49 | <.001 | 0.51  (0.15-0.87) | 62 | 0.65  2.16 | 4.73  4.66 | <.05 | 0.28  (-0.08-0.63) | 62 | 0.65  2.65 | 4.73  4.52 | <.01 | 0.36  (0.003-0.71) |
| PSQ-18**^b^** | 81 | 3.53  3.76 | 0.58  0.44 | <.01 | 0.37  (0.05-0.67) | 81 | 3.53  3.77 | 0.58  0.47 | <.01 | 0.36  (0.05-0.67) | 81 | 3.53  3.69 | 0.58  0.51 | <.05 | 0.23  (-0.08-0.54) |
| CGI**^c^**  _global_ | 95 | 4.26  3.24 | 1.22  1.37 | <.001 | -0.80  (-1.09-[-0.50]) | 95 | 4.26  3.06 | 1.22  1.37 | <.001 | -0.85  (-1.15-[-0.55]) | 95 | 4.26  3.08 | 1.22  1.41 | <.001 | -0.91  (-1.21-[-0.62]) |
| PANSS**^d^**  ^positive^ | 95 | 17.78  13.28 | 6.73  6.50 | <.001 | -0.71  (-1.0-[-0.41]) | 95 | 17.78  12.43 | 6.73  6.35 | <.001 | -0.77  (-1.07-[-0.48]) | 95 | 17.78  12.35 | 6.73  6.25 | <.001 | -0.76  (-1.06-[-0.47]) |
| PANSS**^d^**  ^negative^ | 94 | 13.73  12.82 | 6.58  6.09 | >.05 | -0.16  (-0.45-0.12) | 94 | 13.73  12.39 | 6.58  5.70 | <.05 | -0.21  (-0.49-0.08) | 94 | 13.73  12.01 | 6.58  5.38 | <.01 | -0.26  (-0.55-0.02) |
| PANSS**^d^**  ^general^ | 93 | 31.89  27.12 | 8.95  7.79 | <.001 | -0.52  (-0.82-[-0.23]) | 93 | 31.89  25.41 | 8.95  7.55 | <.001 | -0.61  (-0.91-[-0.32]) | 93 | 31.89  25.03 | 8.95  7.54 | <.001 | -0.60  (-0.90-[-0.31]) |
| PANSS**^d^**  ^total^ | 92 | 63.87  53.47 | 17.52  17.09 | <.001 | -0.62  (-0.91-[-0.32]) | 92 | 63.87  50.42 | 17.52  17.45 | <.001 | -0.70  (-0.99-[-0.40]) | 92 | 63.87  49.57 | 17.52  16.70 | <.001 | -0.70  (-0.99-[-0.40]) |
| GAF**^e^** | 95 | 46.40  55.72 | 13.59  15.95 | <.001 | 0.57  (0.45-1.03) | 95 | 46.40  60.23 | 13.59  17.75 | <.001 | 0.98  (0.68-1.28) | 95 | 46.40  60.76 | 13.59  18.08 | <.001 | 1.03  (0.72-1.33) |

LOCF= Last observation carried forward; *M*=mean; *SD*=standard deviation; Δ^change^=changes in an outcome between assessment timepoints; *p*=.02, significance Level; d=effect size; CI=critical interval.

**^a^**DAI-10, Drug Attitudes Inventory [50, 51].

**^b^**PSQ-18, Patient Satisfaction Questionnaire [52].

**^c^**CGI global, Clinical Global Impression-Schizophrenia Scale global value [54].

**^d^**PANSS (positive, negative, general, total), Positive and Negative Syndrome Scale with subscales positive and negative symptoms, general symptoms and total score [55].

**^e^**GAF, Global Assessment of Functioning Scale [56, 57].

**Table S4.** Clinical symptoms, functioning and psychosocial concepts at baseline and follow-ups: paired t-tests of MI data

|  | Baseline vs. 6-week follow-up | | | | | Baseline vs. 6-month follow-up | | | | | Baseline vs. 12-month follow-up | | | | |
| --- | --- | --- | --- | --- | --- | --- | --- | --- | --- | --- | --- | --- | --- | --- | --- |
| Outcome | *n* | *M* | *SD* | *p* | *d*  (95% CI) | *n* | *M* | *SD* | *p* | *d*  (95% CI) | *n* | *M* | *SD* | *p* | *d*  (95% CI) |
| DAI-10**^a^** | 95 | 0.75  4.13 | 4.10  3.14 | <.001 | 0.65  (0.36-0.94) | 95 | 0.75  2.82 | 4.10  3.70 | <.001 | 0.59  (0.30-0.88) | 95 | 0.75  4.11 | 4.10  3.07 | <.01 | 0.63  (0.33-0.92) |
| PSQ-18**^b^** | 95 | 3.53  3.78 | 0.61  0.45 | <.001 | 0.31  (0.03-0.60) | 95 | 3.53  3.79 | 0.61  0.53 | <.01 | 0.33  (0.04-0.62) | 95 | 3.53  3.77 | 0.61  0.65 | <.05 | 0.30  (0.01-0.58) |
| CGI**^c^**  _global_ | 95 | 4.26  2.83 | 1.22  1.19 | <.001 | -0.92  (-1.21-[-0.62]) | 95 | 4.26  2.57 | 1.22  1.19 | <.001 | -1.04  (-1.35-[-0.74]) | 95 | 4.26  2.72 | 1.22  1.21 | <.001 | -1.00  (-1.30-[-0.70]) |
| PANSS**^d^**  ^positive^ | 95 | 17.78  10.31 | 6.73  3.09 | <.001 | -0.85  (-1.15-[-0.55]) | 95 | 17.78  9.55 | 6.73  3.05 | <.001 | -0.93  (-1.23-[-0.63]) | 95 | 17.78  9.72 | 6.73  3.16 | <.001 | -0.86  (-1.16-[-0.57]) |
| PANSS**^d^**  ^negative^ | 95 | 13.69  12.88 | 6.56  5.47 | >.05 | -0.12  (-0.40-0.17) | 95 | 13.69  11.42 | 6.56  4.14 | <.01 | -0.31  (-0.59-[-0.02]) | 95 | 13.69  11.09 | 6.56  3.86 | <.001 | -0.35  (-0.63-[0.06]) |
| PANSS**^d^**  ^general^ | 95 | 31.88  26.03 | 8.86  6.65 | <.001 | -0.58  (-0.87-[-0.28]) | 95 | 31.88  23.11 | 8.86  4.88 | <.001 | -0.76  (-1.05-[-0.46]) | 95 | 31.88  22.60 | 8.86  4.79 | <.001 | -0.76  (-1.06-[-0.47]) |
| PANSS**^d^**  ^total^ | 95 | 62.59  48.13 | 18.68  13.77 | <.001 | -0.69  (-0.98-[-0.40]) | 95 | 62.59  44.04 | 18.68  10.14 | <.001 | -0.78  (-1.07-[-0.48]) | 95 | 62.59  43.21 | 18.68  9.57 | <.001 | -0.74  (-1.03-[-0.44]) |
| GAF**^e^** | 95 | 46.40  60.71 | 13.59  12.02 | <.001 | 0.91  (0.61-1.21) | 95 | 46.40  65.76 | 13.59  13.30 | <.001 | 1.16  (0.85-1.47) | 95 | 46.40  67.87 | 13.59  13.26 | <.001 | 1.30  (0.99-1.61) |

MI= Multiple imputation; *M*=mean; *SD*=standard deviation; Δ^change^=change in an outcome between assessment timepoints; *p*=.02, significance Level; d=effect size; CI=critical interval.

**^a^**DAI-10, Drug Attitudes Inventory [50, 51].

**^b^**PSQ-18, Patient Satisfaction Questionnaire [52].

**^c^**CGI global, Clinical Global Impression-Schizophrenia Scale global value [54].

**^d^**PANSS (positive, negative, general, total), Positive and Negative Syndrome Scale with subscales positive and negative symptoms, general symptoms and total score [55].

**^e^**GAF, Global Assessment of Functioning Scale [56, 57].

**Table S5.** Subgroup analyses of primary and secondary outcomes for NSIPD group at baseline and follow-ups: paired t-tests ofobserved data.

|  | Baseline vs. 6-week follow-up | | | | | | Baseline vs. 6-month follow-up | | | | | | Baseline vs. 12-month follow-up | | | | | |
| --- | --- | --- | --- | --- | --- | --- | --- | --- | --- | --- | --- | --- | --- | --- | --- | --- | --- | --- |
| Outcome | *n* | M | SD | Δ  ^change^ | *p* | *d*  (95% CI) | *n* | M | SD | Δ  ^change^ | *p* | *d*  (95% CI) | *n* | M | SD | Δ  ^change^ | *p* | *d*  (95% CI) |
| DAI-10^a^ | 29 | 0.62  4.00 | 4.35  3.42 | 3.38 | <.01 | 0.57  (0.04-1.09) | 25 | 0.48  4.16 | 4.48  4.65 | 3.68 | <.01 | 0.63  (0.06-1.20) | 19 | 0.53  4.53 | 4.26  3.99 | 4.00 | <.01 | 0.81  (0.14-1.45) |
| PSQ-18^b^ | 36 | 3.48  3.78 | 0.64  0.44 | 0.3 | <.01 | 0.49  (0.03-0.96) | 33 | 3.54  3.75 | 0.51  0.45 | 0.21 | <.05 | 0.36  (-0.13-0.85) | 25 | 3.66  3.72 | 0.49  0.59 | 0.06 | .55 | 0.13  (-0.43-0.68) |
| CGI^c^  ^global^ | 40 | 4.33  2.98 | 1.16  1.12 | 1.35 | <.001 | -1.08  (-1.55-[-0.61]) | 38 | 4.24  2.79 | 1.20  1.12 | 1.45 | <.001 | -0.90  (-1.37-[-0.43]) | 32 | 4.28  2.63 | 1.25  1.30 | 0.05 | <.001 | -1.33  (-1.88-[-0.79]) |
| PANSS^d^  ^positive^ | 40 | 15.03  10.55 | 5.74  3.49 | 4.48 | <.001 | -0.70  (-1.15-[-0.25]) | 38 | 15.03  9.92 | 5.82  3.98 | 5.11 | <.001 | -0.87  (-1.34-[-0.40]) | 32 | 14.37  8.91 | 5.23  2.57 | 5.46 | <.001 | -0.95  (-1.46-[-0.43]) |
| PANSS^d^  ^negative^ | 39 | 15.33  14.03 | 7.27  6.49 | 1.52 | .16 | -0.22  (-0.66-[-0.23]) | 38 | 14.21  11.97 | 6.94  5.25 | 2.24 | <.05 | -0.36  (-0.81-[-0.10]) | 32 | 15.09  12.03 | 7.68  5.53 | 3.06 | =.01 | -0.45  (-0.94-[-0.05]) |
| PANSS^d^  ^general^ | 40 | 31.85  25.90 | 8.44  6.67 | 5.95 | <.001 | -0.71  (-1.16-[-0.26]) | 38 | 31.42  23.66 | 8.64  6.14 | 7.76 | <.001 | -0.73  (-1.19-[-0.32]) | 32 | 31.75  22.00 | 8.88  5.07 | 9.75 | <.001 | -0.87  (-1.38-[-0.36]) |
| PANSS^d^  ^total^ | 39 | 62.17  50.13 | 16.31  14.69 | 12.04 | <.001 | -0.86  (-1.32-[-0.40]) | 38 | 60.66  45.55 | 16.59  13.10 | 15.11 | <.001 | -0.89  (-1.36-[-0.41]) | 32 | 61.22  42.93 | 17.08  10.86 | 18.29 | <.001 | -0.97  (-1.49-[-0.45]) |
| GAF^e^ | 39 | 46.69  59.41 | 13.31  14.86 | 12.72 | <.001 | 1.01  (0.54-1.48) | 38 | 46.79  64.58 | 12.18  15.56 | 17.79 | <.001 | 1.28  (0.78-1.78) | 31 | 47.26  66.13 | 12.74  15.83 | 18.87 | <.001 | 1.65  (1.08-2.23) |

*M*=mean; *SD*=standard deviation; Δ^change^=change in an outcome between assessment timepoints; *p*=.02, significance Level; d=effect size; CI=critical interval; NSIPD= Non substance-induced psychotic disorder.

**^a^**DAI-10, Drug Attitudes Inventory [50, 51].

**^b^**PSQ-18, Patient Satisfaction Questionnaire [52].

**^c^**CGI global, Clinical Global Impression-Schizophrenia Scale global value [54].

**^d^**PANSS (positive, negative, general, total), Positive and Negative Syndrome Scale with subscales positive and negative symptoms, general symptoms and total score [55].

**^e^**GAF, Global Assessment of Functioning Scale [56, 57].

**Table S6.** Subgroup analyses of primary and secondary outcomes for SIPD group at baseline and follow-ups: paired t-tests of observed data

|  | Baseline vs. 6-week follow-up | | | | | | Baseline vs. 6-month follow-up | | | | | | Baseline vs. 12-month follow-up | | | | | |
| --- | --- | --- | --- | --- | --- | --- | --- | --- | --- | --- | --- | --- | --- | --- | --- | --- | --- | --- |
| Outcome | *n* | *M* | *SD* | Δ  ^change^ | *p* | *d*  (95% CI) | *n* | *M* | *SD* | Δ  ^change^ | *p* | *d*  (95% CI) | *n* | *M* | *SD* | Δ  ^change^ | *p* | *d*  (95% CI) |
| DAI-10^a^ | 19 | 2.21  4.63 | 4.66  3.40 | 2.42 | <.02 | 0.55  (-0.1-1.20) | 15 | 1.20  0.80 | 5.54  3.84 | 0.40 | <.77 | -0.07  (-0.78-0.65) | 14 | 0.71  2.43 | 6.06  3.94 | 1.72 | .30 | 0.25  (-0.50-0.99) |
| PSQ-18^b^ | 23 | 3.56  3.91 | 0.64  0.42 | 0.35 | .06 | -0.35  (-0.23-0.94) | 20 | 3.49  3.89 | 0.64  0.51 | 0.40 | <.05 | 0.41  (-0.22-1.03) | 18 | 3.53  3.75 | 0.70  0.54 | 0.22 | .37 | 0.19  (-0.47-0.84) |
| CGI^c^  ^global^ | 25 | 4.36  2.64 | 1.29  1.38 | 1.72 | <.001 | -1.05  (-1.64-[-0.46]) | 22 | 4.23  2.32 | 1.34  1.32 | 1.91 | <.001 | -1.09  (-1.73-[-0.46]) | 23 | 4.17  2.57 | 1.27  1.59 | 1.60 | <.001 | -0.97  (-1.57-[-0.35]) |
| PANSS^d^  ^positive^ | 26 | 19.42  10.04 | 6.84  3.57 | 9.38 | <.001 | -1.03  (-1.61-[-0.46]) | 21 | 20.14  8.95 | 7.88  2.92 | 11.19 | <.001 | -0.92  (-1.55-[-0.28]) | 23 | 19.87  10.48 | 7.25  5.23 | 9.39 | <.001 | -0.85  (-1.46-[-0.25]) |
| PANSS^d^  ^negative^ | 25 | 12.52  11.12 | 6.36  5.88 | 1.40 | .37 | -0.18  (-0.73-[-0.38]) | 21 | 12.85  10.66 | 6.78  4.68 | 2.19 | .27 | -0.21  (-0.82-0.39) | 23 | 12.61  9.82 | 6.38  3.20 | 2.79 | .09 | -0.29  (-0.87-0.29]) |
| PANSS^d^  ^general^ | 25 | 34.08  25.84 | 11.23  9.52 | 8.24 | <.01 | -0.63  (-1.20-[-0.07]) | 21 | 33.52  21.85 | 11.96  5.41 | 11.67 | <.01 | -0.68  (-1.30-[-0.06]) | 23 | 33.61  23.13 | 10.53  7.18 | 10.48 | <.01 | -0.62  (-1.21-[-0.33) |
| PANSS^d^  ^total^ | 25 | 65.69  45.58 | 20.97  18.80 | 20.11 | <.001 | -0.83  (-1.40-[-0.26]) | 21 | 66.52  41.47 | 22.25  11.29 | 25.05 | <.001 | -0.74  (-1.37-[-0.12]) | 23 | 66.09  43.43 | 21.56  14.07 | 22.66 | <.01 | -0.66  (-1.25-[-0.07]) |
| GAF^e^ | 25 | 47.20  62.76 | 14.08  13.89 | 15.56 | <.001 | 0.91  (0.33-1.49) | 22 | 47.64  68.64 | 15.48  17.81 | 21.00 | <.001 | 1.12  (0.48-1.76) | 22 | 49.73  70.18 | 15.07  19.34 | 20.45 | <.01 | 1.00  (0.37-1.62) |

*M*=mean; *SD*=standard deviation; Δ^change^=change in an outcome between assessment timepoints; *p*=.02, significance Level; d=effect size; CI=critical interval; SIPD=Substance-induced psychotic disorder.

**^a^**DAI-10, Drug Attitudes Inventory [50, 51].

**^b^**PSQ-18, Patient Satisfaction Questionnaire [52].

**^c^**CGI global, Clinical Global Impression-Schizophrenia Scale global value [54].

**^d^**PANSS (positive, negative, general, total), Positive and Negative Syndrome Scale with subscales positive and negative symptoms, general symptoms and total score [55].

**^e^**GAF, Global Assessment of Functioning Scale [56, 57].
